# Implementation of patient safety strategies in European hospitals

**DOI:** 10.1136/qshc.2008.029413

**Published:** 2009-01-26

**Authors:** R Suñol, P Vallejo, O Groene, G Escaramis, A Thompson, B Kutryba, P Garel

**Affiliations:** 1 Avedis Donabedian Institute, Autonomous University of Barcelona, and CIBER Epidemiology and Public Health (CIBERESP), Barcelona, Spain; 2 Biostatistics Unit, Department of Public Health, University of Barcelona, and CIBER Epidemiology and Public Health (CIBERESP), Barcelona, Spain; 3 School of Social and Political Studies, University of Edinburgh, Edinburgh, Scotland; 4 National Centre for Quality Assessment in Health Care (NCQA), Krakow, Poland; 5 European Hospital and Healthcare Federation (HOPE), Brussels, Belgium

## Abstract

**Context:**

This study is part of the Methods of Assessing Response to Quality Improvement Strategies (MARQuIS) research project on cross-border care, investigating quality improvement strategies in healthcare systems across the European Union (EU).

**Aim:**

To explore to what extent a sample of acute care European hospitals have implemented patient safety strategies and mechanisms and whether the implementation is related to the type of hospital.

**Methods:**

Data were collected on patient safety structures and mechanisms in 389 acute care hospitals in eight EU countries using a web-based questionnaire. Subsequently, an on-site audit was carried out by independent surveyors in 89 of these hospitals to assess patient safety outputs. This paper presents univariate and bivariate statistics on the implementation and explores the associations between implementation of patient safety strategies and hospital type using the χ^2^ test and Fisher exact test.

**Results:**

Structures and plans for safety (including responsibilities regarding patient safety management) are well developed in most of the hospitals that participated in this study. The study found greater variation regarding the implementation of mechanisms or activities to promote patient safety, such as electronic drug prescription systems, guidelines for prevention of wrong patient, wrong site and wrong surgical procedure, and adverse events reporting systems. In the sample of hospitals that underwent audit, a considerable proportion do not comply with basic patient safety strategies—for example, using bracelets for adult patient identification and correct labelling of medication.

As a result of landmark publications on patient safety^1^ ^2^ and subsequent advocacy work and research, considerable attention has focused on improving patient safety and protecting patients. At the international level, this includes the Global Patient Safety Alliance launched by the World Health Organization (WHO) and the development of patient safety indicators within the Organisation for Economic Co-operation and Development (OECD) Health Care Quality Indicator Project.^3^ ^4^ At the European level, the Safety Improvement for Patients In Europe (SIMPATIE) project aims to establish a common European set of vocabulary, indicators, and internal and external instruments for improvement of safety in healthcare.^5^ Another European Union (EU) project, the European Network for Patient Safety (EUNETPAS), aims to establish an umbrella network of all EU member states and stakeholders to encourage and enhance collaboration in the field of patient safety.^6^ At the national level, several European countries have launched studies on the incidence of adverse events or initiated projects to improve patient safety.^7^ ^8^ Finally, several national hospital accreditation schemes also collect data on accomplishment of their safety standards. However, despite the broad range of actions to improve patient safety little information is available on the current implementation of patient safety strategies in European hospitals.

This study was carried out within the Methods of Assessing Response to Quality Improvement Strategies (MARQuIS) project. The objectives of the MARQuIS project are to describe and compare different quality improvement policies and strategies in healthcare systems across the member states of the EU, and to consider their potential use when patients cross borders to receive healthcare. This research was intended to enable an evaluation of the need for and development of formal quality procedures at the EU level for healthcare services. The objective of this paper was to explore the implementation of patient safety strategies in European hospitals and assess whether implementation was associated with type of hospital.

## METHODS

We conducted an observational study in acute care hospitals in eight European countries (Belgium, Czech Republic, France, Ireland, Netherlands, Poland, Spain and United Kingdom). Data for this study was collected in two independent phases: the first phase consisted of a self-administered, cross-sectional survey on quality improvement strategies in a sample of European hospitals. We randomly selected acute hospitals with more than 100 beds that provided care for at least two out of the three conditions covered in the study (acute myocardial infarction, deliveries and appendicitis). Data were collected with the MARQuIS questionnaire, a 199 issues/questions (about 500 items) data gathering tool. The answers to this questionnaire were self-reported through a secured internet-based application from April to August 2006. Methods and characteristics of the questionnaire, and the profile of responding hospitals, have been described elsewhere in this supplement.^9^

The second phase consisted of an on-site external assessment in a sample of hospitals that answered the questionnaire to test the reliability of the self-reported responses to the questionnaire and to collect detailed evidence of the institutionalisation of quality and safety and the use of different patient safety strategies. A purposeful sample was defined based on the level of maturity of the quality improvement system as determined by a selection of questions from the MARQuIS questionnaire.^10^ Data were collected during a 2-day on-site external assessment carried out by independent and specifically trained surveyors from January to April 2007. A specific instrument was developed for data collection (the MARQuIS audit tool), containing a set of 341 items. For the development of the data-gathering tools we carried out a literature research and consultations with experts. In addition, within the MARQuIS project, a separate research study aimed at identifying particular quality and safety requirements for cross-border patients.^11^ A detailed description of the methods of the external assessment have been discussed elsewhere in this supplement.^12^

For the analysis presented in this paper we distinguished three domains:

- Patient safety strategies: hospital-wide responsibilities regarding patient safety management, including planning for safety, designation of responsibilities and reporting, and implication of leadership plans.
- Patient safety mechanisms: systems put in place by management that have proven to increase patient safety, such as systems for adverse event reporting or standardised number of drugs.
- Patient safety outputs: instead of *outcomes* that we were not able to collect for this study we used intermediate *outputs* for patient safety strategies, based on direct observation during on-site audit.

The presentation of data on the implementation of patient safety strategies is based on the survey results, while the presentation of data for patient safety mechanisms is based on both the survey and the audit results. We calculated compliance with patient safety strategies as the percentage of positive responses out of the total number responses to each of the items. For those items in the audit study with ordinal response scales we aggregated two positive categories (exceptional and extensive compliance). For each domain we present the implementation in the total sample and stratified by type of hospital (university hospital, general hospital with residency training, general hospital without residency training). To assess associations between implementation of patient safety strategy, mechanisms and outputs and type of hospital we computed the χ^2^ statistic and Fisher exact test. As the sample size of the audit did not allow generalisation of results, we have not reported data at country level.

## RESULTS

A total of 389 hospitals from eight EU countries responded to the MARQuIS questionnaire. Of these, 89 underwent an in-depth audit. Characteristics of the participating hospitals are presented in table 1.

**Table 1:** Hospital characteristics

Characteristics	Hospital survey	Hospital audit	p Value (χ^2^ test)
Ownership			0.325
Public	297 (80.7)	72 (81.8)	
Private not-for-profit	37 (10.1)	5 (5.7)	
Private for-profit	34 (9.2)	11 (12.5)	
Type of hospital			0.739
University hospital	85 (23.5)	17 (19.8)	
General residency training	177 (48.9)	43 (50.0)	
General non-teaching	100 (27.6)	26 (30.2)	
Hospital beds			0.822
<200	63 (19.1)	16 (18.6)	
200–399	98 (29.7)	21 (24.4)	
400–599	64 (19.4)	21 (24.4)	
600–799	41 (12.4)	13 (15.1)	
800–999	22 (6.7)	6 (7.0)	
>999	42 (12.7)	9 (10.5)	
Total	389	89	

Participating hospitals were mainly under public ownership and only a small proportion of hospitals were under private ownership, either for-profit or not-for-profit. Half of the hospitals could be described as general hospitals with residency training and about a quarter each as university hospitals and general non-teaching hospitals. The size of the hospitals, as assessed in terms of the number of beds, differed considerably and ranged from small community hospitals with fewer than 200 beds to large university hospitals with more than 1000 beds. For all characteristics presented here, the data in the audit sample matched the data in the questionnaire sample of hospitals.

The analysis of structures and plans for patients’ safety and activities performed by hospitals are reported in table 2. With regard to structures and plans for patient safety, overall, we observed a ceiling effect; almost all hospitals reported having these structures and systems in place. It needs to be taken into consideration that these results were based on self-reported questionnaire and might have been subject to social desirability bias. On the one hand, traditional patient safety responsibilities, such as focus on infections and antibiotics, seemed to be better developed than the more recent focus on patient safety as a generic function that integrates these responsibilities. On the other hand, the data suggested that leadership could be improved substantially: neither the governing boards nor the clinical committees seemed to systematically receive reports on complications and incidence/adverse events. For all items, except responsibilities for blood transfusion, we did not detect any significant difference in implementation rate by type of hospital.

**Table 2:** Implementation of patient safety structures, responsibilities and reporting

Structure, responsibility and reporting	Total yes (%)	University	General with residency training	General non-teaching	p Value
Structure and plan					
Aims and mission include patient safety	338 (96.8)	71 (93.4)	161 (98.2)	85 (96.6)	0.179†
Designated responsibilities for:					
Patient safety	255 (74.6)	58 (74.4)	120 (75.0)	57 (67.9)	0.467*
Hospital infections	349 (98.9)	80 (100.0)	161 (98.2)	88 (100.0)	0.437†
Blood transfusion	321 (94.4)	78 (98.7)	152 (96.2)	73 (88.0)	0.007†
Antibiotics	326 (93.9)	79 (98.8)	151 (93.8)	77 (90.6)	0.060†
Decubitus	295 (87.0)	62 (83.8)	143 (89.9)	70 (81.4)	0.142*
Clinical waste management	332 (95.4)	74 (93.7)	153 (95.0)	84 (96.6)	0.709†
Periodic reports on:					
Patient safety	199 (92.1)	47 (92.2)	88 (89.8)	48 (94.1)	0.720†
Hospital infections	296 (98.7)	70 (100.0)	132 (99.2)	77 (96.3)	0.147†
Blood transfusion	258 (93.5)	66 (97.1)	115 (92.0)	62 (91.2)	0.322†
Antibiotics	258 (94.5)	65 (97.0)	114 (94.2)	64 (92.8)	0.529†
Decubitus	216 (88.5)	47 (88.7)	101 (89.4)	56 (88.9)	1.000†
Clinical waste management	250 (89.0)	59 (95.2)	109 (87.2)	65 (84.4)	0.111†
Implication of leadership—governing board receives:					
Report on complication registration	160 (50.5)	41 (60.3)	69 (44.2)	39 (52.0)	0.079*
Incidence/adverse events	209 (63.9)	43 (60.6)	94 (60.3)	58 (71.6)	0.196*
Implication of leadership—clinical committee receives:					
Report on complication registration	169 (63.3)	39 (69.6)	71 (56.3)	45 (66.2)	0.165*
Incidence/adverse events	198 (71.0)	38 (64.4)	89 (67.9)	56 (77.8)	0.201*

*χ^2^ test; †Fisher exact test.

Moving from policies and structures (table 2) to concrete mechanisms and activities (table 3), we found that the rates of implementation seemed to drop, even though these data were still based on self-reported questionnaire. Only the item “standardised and limited number of drugs” suggested a ceiling effect, for the remaining items implementation was considerably lower. The data suggest that systems for reporting and analysing adverse events were more developed at departmental than at hospital level. Despite the high frequency of wrong patient, wrong surgery reports in the literature on adverse events, only half of the hospitals had specific guidelines in place to reduce such incidents. Moreover, electronic drugs prescription systems were in place in fewer than half of the hospitals. For these mechanisms or activities we did not detect a difference by type of hospital.

**Table 3:** Patient safety mechanisms and activities (survey)

Mechanism or activity	Total yes (%)	University	General with residency training	General non-teaching	p Value (χ^2^ test)
Standardised and limited number of drugs	318 (91.9)	74 (94.9)	149 (92.0)	79 (89.8)	0.478
System for reporting and analysis of adverse events available in departments	170 (63.9)	32 (56.1)	85 (62.0)	48 (78.7)	0.101
System for reporting and analysis of adverse events available in the hospital	174 (50.7)	36 (46.2)	69 (43.9)	54 (60.7)	0.231
Guideline/protocol for the prevention of wrong patient/wrong surgical procedure	129 (47.1)	32 (53.3)	60 (45.5)	30 (42.9)	0.459
Electronic drug prescription system	138 (39.8)	38 (48.7)	60 (37.3)	34 (38.2)	0.215

Table 4 shows the data on the hospital audit conducted in 89 hospitals. Hospital-wide assessment was conducted for some of the items whereas others were assessed in distinct departments. At hospital level, the item with the highest compliance was the use of identification bracelets for newborns. Newborn resuscitation equipment was available in about 90% of the hospitals, but given that this is basic and live-saving equipment, this rate should not be considered as high. Surprisingly, university hospitals reported lower rates for the availability of this equipment, although the difference was not significant (Fisher exact test) because of the small sample size. The two outputs called “Data show improvement in patient safety/medication safety after committee intervention” aimed to evaluate whether hospitals monitored their safety interventions and could show concrete improvements in relation to some of the initiatives that had taken place. To be accepted as such, improvements needed to have been recorded before the evaluation and the measurement system that was used for its assessment made explicit. For our study, any type of improvement was acceptable, including changes in ward structure, increase in use of bracelets, etc.

**Table 4:** Patient safety outputs based on hospital audit

	Total yes (%)	University	General with residency training	General non-teaching	p Value
Hospital-wide:					
Newborn identification (ID)	74 (96.1)	12 (92.3)	39 (95.1)	20 (100.0)	0.747†
Newborn resuscitation equipment available	67 (88.2)	10 (76.9)	37 (90.2)	17 (89.5)	0.440†
Access to neonatal nursery controlled by door locks	54 (73.0)	10 (76.9)	27 (69.2)	14 (73.7)	0.848*
Medication dispensed from pharmacy is fully labelled	44 (62.0)	9 (64.3)	28 (75.7)	7 (41.2)	0.048*
Data show improvement in patient safety after committee intervention	24 (27.9)	3 (18.8)	11 (26.8)	7 (26.9)	0.798*
Data show improvement in medication safety after committee intervention	22 (25.0)	6 (35.3)	7 (16.7)	7 (26.9)	0.276*
Department-level:					
High-risk drugs are stored separately:					
Maternity	56 (74.7)	8 (66.7)	32 (80.0)	13 (65.0)	0.386*
Medicine	67 (79.8)	13 (76.5)	33 (80.5)	18 (78.3)	0.938†
Surgery	62 (73.8)	12 (75.0)	27 (69.2)	20 (76.9)	0.774*
Drugs storage locked:					
Maternity	52 (70.3)	9 (69.2)	24 (61.5)	16 (84.2)	0.215*
Medicine	56 (68.3)	10 (58.8)	29 (70.7)	14 (66.7)	0.679*
Surgery	57 (67.1)	12 (70.6)	23 (59.0)	19 (76.0)	0.344*
Alcohol rub dispensers:					
Maternity	49 (63.6)	8 (61.5)	25 (61.0)	13 (65.0)	0.954*
Medicine	56 (66.7)	9 (52.9)	27 (65.9)	17 (73.9)	0.385*
Surgery	54 (62.1)	9 (52.9)	26 (63.4)	16 (61.5)	0.755*
Adult patient ID:					
Maternity	35 (47.3)	6 (50.0)	20 (50.0)	8 (42.1)	0.840*
Medicine	21 (25.3)	4 (23.5)	9 (22.5)	5 (21.7)	0.991*
Surgery	25 (29.1)	5 (29.4)	10 (24.4)	8 (30.8)	0.831*

*χ^2^ test; †Fisher exact test.

At departmental level, compliance with patient safety outputs included in this study was only average and the data suggested sufficient room for improvement, for example with regard to provision of alcohol rub dispensers to improve hand hygiene or the use of identification bracelets for adult patients. Moreover, there did not seem to be a difference in compliance rates across departments, except for adult patient identification by using bracelets, which was better complied with in maternity departments. Similar to the results to the questionnaire, we could not differentiate compliance with patient safety output by type of hospital, either at hospital or department level.

## DISCUSSION

Our study provides information about the implementation of safety strategies and mechanisms in a sample of 389 European hospitals, based in self-reported data and more detailed information about outputs in 89 hospitals based on an external audit.

There are a number of limitations of the study that need to be mentioned. First, although of great policy concern, it was not the aim of this study to present generalisable findings at country level with regard to the implementation of patient safety strategies. In the context of cross-border care, all hospitals potentially deliver services to EU citizens and an evaluation of country differences is thus secondary. Moreover, the differences in the rates of response to the questionnaire in different European countries and the sample size of the audit did not allow further stratification and generalisation. A second limitation of the study is that it did not include outcome data related to patient safety, owing to the limitations of the overall research objectives, differences in data coding and availability in the participating countries, and generally a low frequency of patient safety events in individual hospitals. Instead, we focused on the patient safety outputs that reflected whether systems were fully implemented, thus providing a criterion regardless of hospital volume or case-mix.

This study has shown that European hospitals have already developed the structure and the plans needed to manage patient safety. The designation of responsible people for the different areas of patient safety seems widely developed, although governing bodies and clinical representative committees are not frequently involved in the analysis of safety monitoring data. This may mean that safety leadership is not yet fully incorporated into the management of the organisations and safety initiatives could function somehow as a parallel process. Regarding the main opportunities identified for improvement related to mechanisms to promote patient safety, it is worth noting that only 40% of the hospitals included on this study had an electronic drug prescription system, even though these systems have proved to be associated with the reduction of prescription errors^13^^–^15; a study carried out in the USA in 2004 found it was available in almost 70% of the hospitals.^16^ The implementation of a guideline or protocol for the prevention of wrong patient, wrong site and wrong surgical procedure, currently available in fewer than half of the hospitals, should also be reinforced. The need for this protocol can be illustrated by the fact that surgery on the wrong site was the most frequent type of adverse event in an analysis by The Joint Commission in March 2008 on 4977 sentinel events, accounting for 13.1% of all adverse events.^17^ Therefore, it appears that the implementation of adverse events reporting systems in hospitals needs to be further promoted. Although one of the issues promoted by the World Alliance for Patient Safety from WHO^18^ is focused on the development of these systems and some of the countries involved in this study have already developed systems at a national level,^19^^–^21 only 51% of the hospitals participating in the study reported having this system available.

We did not detect any differences in the implementation of patient safety strategies, mechanisms and outputs by hospital type, with the exception of implementation of policies for blood transfusion. This shows that the patient safety issues assessed in this paper are generic in nature and should thus be implemented irrespective of hospital type.

Despite the ceiling effect observed for some of the items, it needs to be pointed out that that we assessed basic patient safety strategies, mechanisms and outputs for which a growing evidence base demonstrates effectiveness in reducing adverse events and improving safety. For example, patient identification by bracelet, one of the goals of the WHO Global Patient Safety Alliance, was only poorly complied with by the hospital in our sample.^22^ Other areas to be targeted for quality improvement are correct labelling of medication dispensed from the pharmacy and implementation of unit-dose systems for medications.

## CONCLUSION

Based on our findings it seems that patient safety structures, activities and outputs in our sample are less developed than those reported in US studies though more studies need to be done to allow a generalization to European level. It seems important to recommend further efforts to implement patients’ safety systems in European hospitals and to promote further studies to understand its development, in particular targeting those areas where currently compliance is still low.
